# Posttraumatic stress disorder, depressive and cardiovascular disease symptoms among young patients receiving medical treatment in a heart centre: A cross-sectional study

**DOI:** 10.51866/oa.557

**Published:** 2024-08-06

**Authors:** Yoke Yong Chen, Siti Raudzah Ghazali, Asri Said

**Affiliations:** 1 BSc (Psychology), MSc (Clinical Psychology), PhD (Health Psychology), Department of Psychological Medicine, Faculty of Medicine and Health Science, Universiti Malaysia Sarawak, Jalan Datuk Mohammad Musa, Kota Samarahan, Sarawak, Malaysia. Email: yychen@unimas.my; 2 BSc (Psychology), MSc (Clinical Psychology), PhD (Clinica sychology), Department of Psychological Medicine, Faculty of Medicine and Health Science, Universiti Malaysia Sarawak, Jalan Datuk Mohammad Musa, Kota Samarahan, Sarawak, Malaysia.; 3 MD,MMED (Int Med), FNHAM, Faculty of Medicine and Health Science, Universiti Malaysia Sarawak, Jalan Datuk Mohammad Musa, Kota Samarahan, Sarawak, Malaysia.

**Keywords:** PTSD, Cardiovascular disease, Trauma, Depression

## Abstract

**Introduction:**

Exploring the connections between traumatic experiences and subsequent health outcomes is vital for informing clinical practices and public health policies. The study aimed to investigate the relationship between lifetime trauma exposure and posttraumatic stress disorder (PTSD), depressive and cardiovascular disease (CVD) symptoms.

**Methods:**

A total of 171 patients who received treatment in a local heart centre were included in this study. Several questionnaires such as the Life Event Checklist-5, Posttraumatic Stress Disorder Checklist for DSM-5 and Patient Health Questionnaire-9 were used to measure their traumatic experiences and PTSD and depressive symptoms, respectively. Physiological measures were also examined. Data were analysed using SPSS.

**Results:**

The chi-square test showed significant differences in the percentage of reported PTSD symptoms among the patients with CVD (24.0%), patients with kidney disease (4.3%) and patients with other health problems (7.1%). The patients with CVD reported having a significantly higher percentage of PTSD and depressive symptoms than the patients with other medical conditions. The patients with CVD who reported having PTSD symptoms had significant systolic blood pressure (SBP) and heart rate changes compared to the patients who did not. The patients who reported PTSD symptoms had a significantly shorter sleep duration than their counterparts. The SBP and diastolic blood pressure differed significantly between the patients with and without PTSD symptoms.

**Conclusion:**

Earlier detection, prevention and intervention related to trauma exposure and PTSD symptoms are suggested to reduce the CVD risk.

## Introduction

In Malaysia, cardiovascular and pulmonary diseases represented 7738 admissions or 16.09% of the total mortality in 2009,^1^ increasing to 28.1% in 2012.^2^ The Malaysia National Strategic Plan for Non-Communicable Disease^3^ lists stress as a cardiovascular disease (CVD) risk factor. Adverse childhood experiences intensify posttraumatic stress disorder (PTSD) symptoms in adulthood,4,5 a psychiatric disorder resulting from life-threatening trauma. PTSD includes symptoms like anxiety, hopelessness with a negative worldview and avoidant behaviours.^4,5^ impacting both mental and physical health, particularly heart disease.^4-6^

Individuals with trauma exposure accompanied by chronic stress and PTSD often exhibit prolonged unhealthy stress responses.^7^ Individuals with PTSD following acute cardiovascular episodes face increased risk of subsequent CVD events and mortality.^4-6^ Increased trauma exposure and/or PTSD have more adverse cardiovascular-related outcomes and higher CVD-related mortality rates.^5,6^ Vaccarino et al.^8^ highlighted a strong association between PTSD and coronary heart disease. They found that twins with PTSD showed a higher incidence of coronary heart disease (90%) and a more compromised myocardial perfusion than twins without PTSD even after counting for established heart disease risk factors. They concluded that shared environmental and genetic factors do not fully explain this association.^8^ Among military personnel and veterans, 8.3% experience illness-induced PTSD, particularly in conjunction with heart disease and the presence of stroke doubles the prevalence of non-life-threatening physical health conditions.^9^

Consistent with the bidirectional association between CVD and PTSD, literature suggests a similar association with depression. Depression is more prevalent among patients with CVD patients than the general population, leading to higher mortality rates and poorer health outcomes.^10,11^ However, most previous studies have focused on middle-aged populations.

Traumatic exposure and PTSD negatively impact mental and physical health. However, previous studies often focus on men or military veterans with disproportionately high rates of obesity, diabetes, hypertension, psychological disorders and CVD, limiting generalizability. Most research targets the older population and specific trauma types, overlooking other traumatic events.^8-10^ Young patients undergoing medical treatment, particularly for chronic conditions like CVD, face unique challenges compared to older adults.^5,6^ Their developmental stage and the long-term impact of medical interventions may heighten susceptibility to trauma and stress affecting cardiovascular health. Investigating the association between PTSD and CVD in this population is critical for early risk identification and preventive measures. However, literature gaps persist in understanding trauma exposure and PTSD among young CVD patients. Our study aimed to establish this association, hypothesizing that a high prevalence of PTSD symptoms and significant physiological differences between traumatised and non- traumatised young CVD patients. This endeavour is crucial for informing effective prevention and treatment strategies.

## Methods

### Participants

A total of 171 patients were recruited from Sarawak General Hospital Heart Centre with a mean age of 30.1 years (Standard Deviation(SD)=11.24) and 52% were men. Seventy-five (43.9%) patients received an official CVD diagnosis. Individuals who registered in local heart centre, were aged 18-40 years and were willing to participate in the study were included. They were randomly selected from the registration files. Conversely, patients who were at high risk or undergoing surgery on the same day of data collection were excluded. Table 1 presents participants’ detailed demographic characteristics.

**Table 1:** Descriptive characteristics of the participants (N=171).

Characteristics	Clinical sample
**Age (M, SD), year**	30.1 (11.24)
**Sex (n, %)**	
Female	82 (48)
Male	89 (52)
**Ethnicity (n, %)**	
Malay	81 (47.4)
Iban	47 (27.5)
Chinese	25 (14.6)
Bidayuh	9 (5.3)
Others	9 (5.3)
**Disease diagnosed (n, %)**	
Healthy^*^	46 (26.9)
Heart disease	75 (43.9)
Heart disease since infancy	13 (7.6)
Kidney disease	23 (13.5)
Others	14 (8.2)
**Multiple trauma experiences (n, %)**	54 (31.6)
Duration^**^ (M, SD), year	4.37 (5.9)

* Healthy individuals included those registered in the local heart centre with some cardiovascular disease (CVD) complaints or those referred by general practitioners. However, upon investigation and examination, they needed only to continue self-monitoring without further follow-up or to be prescribed medication.

** Duration of traumatic experience = year of study administration - year of trauma exposure.

### Measures

Demographic variables including age, sex and ethnicity were collected.

**Trauma exposure**

The participants were instructed to complete the Life Event Checklist-5 (LEC-5),^12^ which contains 17 items on traumatic and negative life events. Each question asks whether individuals have either direct or indirect exposure to the listed potentially traumatic events. In this study, five items derived from the literature review and pilot study were added to the original 17 items including near drowning, robbery, parental separation, persecution/humiliation and childhood neglect. The Cronbach’s alpha value of the LEC-5 was 0.73.

**PTSD**

The Posttraumatic Stress Disorder Checklist for DSM-5 (PCL-5)^13^ is a self-administered questionnaire with 20 items corresponding to the DSM-5 PTSD symptoms. Generally, individuals respond to each item on a 5-point Likert scale to reflect the level of distress associated with each symptom, generating a total score ranging from 0 to 80. A cut-off of 33 is utilised to determine the absence and presence of PTSD.^14^ In the present study, the PCL-5 showed good internal consistency (α=0.91).

**Depression**

The Patient Health Questionnaire-9 (PHQ-9)^15^ is a nine-item self-report questionnaire used to detect the presence of depressive symptoms in the past seven days. Each item is scored on a 4-point scale, yielding a total score ranging from 0 to 27. A total score of 0-4 is interpreted as *no depressive symptoms,* 5-9 as *mild depression,* 1014 as *moderate depression,* 15-19 as *moderately severe depression* and ≥20 as *severe depression.* The Malay-version PHQ-9 was validated in a Malaysian primary care clinic.^16^ The Cronbach’s alpha value of the PHQ-9 was 0.81.

**CVD and other physical health problems**

The medical records of the participants were checked to obtain their health status.

**Smoking, alcohol drinking and sleeping duration**

An adapted US National Health Interview Survey Checklist^17^ was used among the participants who had ever engaged in health- related behaviours (e.g. cigarette smoking, alcohol drinking and sleeping duration) and experienced a series of symptom-based conditions (e.g. chronic headache and chronic low back pain). Smoking intensity was categorized as 0 (never smoked), 1 (1-10 cigarettes/day), 2 (11–20 cigarettes/day), 3 (>20 cigarettes/day), or 4 (former smoker). Heavy alcohol use was defined as consuming five or more drinks (>3000 mL for 2%–5% alcohol) on five or more occasions weekly over the past 30 days. Moderate drinking included one drink per day for women and two for men. Participants also reported their nightly sleep duration.

**Physical activities**

The participants were asked about the frequency and intensity of their physical activity in a week. The agreement between self-reported and physician-diagnosed health issues and physical activities was strong, resulting in limited bias for research purposes in studying the association between mental and physical health.^18^ For the full study sample, the Cronbach’s alpha value of the adapted US National Health Interview Survey Checklist in the present study was 0.72.

**Body mass index (BMI)**

BMI was measured using the participant’s height and weight via a stadiometer and weighing scale available at the hospital. It is calculated as kilograms per square metre. A BMI of 25.0-29.9 kg/m^2^ indicates overweight; ≥30 kg/m^2^, obesity; <18.5 kg/m^2^, underweight; and 18.5-24.9 kg/m^2^, normal weight.

**Physiological measures**

Blood pressure was measured using a blood pressure machine provided by the hospital at three time points: before participants completed questionnaires (T1), when the participants completed traumatic events and PCL-5 questionnaires (T2), and after completing all questionnaires (T3). Participants were informed on avoiding blood pressure changes during questionnaire completion.

### Data collection

Patients aged 18-45 years were randomly selected from registration files after obtaining consent from doctors or nurses and participants. All participants provided signed consent and were informed about their rights, potential risks and confidentiality. Procedures and timing of measuring their physiological responses were explained. Demographic information was voluntarily completed by all participants. Participants completed the questionnaires on their traumatic history, mental and physical health history, substance use habits (i.e. alcohol drinking, cigarette smoking), sleeping duration, and physical activities.

### Translation

For this study, all instruments were translated into the Malay language (Bahasa Malaysia) and were back-translated by two academicians who were experts in both English and Malay languages. The content validity and reliability of the translated versions were evaluated and tested in a pilot study.

### Data analysis

Descriptive statistics (frequencies, percentages) were used to present variables including trauma exposure prevalence, the time interval between trauma and assessment, health symptoms, PTSD scores, and BMI. Differences in the physiological measures (blood pressure, heart rate (HR), BMI) and unhealthy behaviours between the participants with and without traumatic experience and PTSD symptoms were evaluated using independent t-test for continuous variables and Pearson chi-square test for dichotomous variables. A P-value of <0.05 was considered significant.

## Results

### Descriptive analysis of trauma exposure and PTSD symptoms

Approximately 83.6% (n=l43) of the participants reported at least one exposure to trauma, while 25.7% exhibited PTSD symptoms. The participants were most commonly exposed to transportation accidents (50.3%), followed by natural disasters (28.1%) and near drowning (22.2%). Table 2 presents the detailed traumatic exposures of the participants. The chi-square test showed significant differences in the percentage of reported PTSD symptoms [X^2^_(1)_=15.56,P=0.004] among the participants with CVD (24.0%), kidney disease (4.3%) and other health problems (7.1%).

**Table 2:** Descript ive characteristics of trauma exposure (N=171).

Trauma exposure	Frequency	Percentage
Transportation accident	86	50.3
Natural disaster	48	28.1
Near drowning	38	22.2
Humiliation/bullying	22	12.9
Fire	19	11.1
Serious accident	19	11.1
Physical assault	18	10.5
Robbery or burglary	11	6.4
Serious self-caused injury	10	5.8
Childhood neglect	7	4.1
Weapon-related assault	5	2.9
Captivity	3	1.8
Sexual assault	2	1.2
Rape	1	0.6
Others	14	8.2

### Descriptive analysis of depressive symptoms

Of the participants, 10.6% reported having moderate-to-severe depressive symptoms. The chi- square test showed significant sex differences in the percentage of reported depressive symptoms [χ^2^_(4)_ =15.00,P=0.005], with the women (18.5%) reporting more depressive symptoms than the men (3.3%) (Table 3). There was a significant difference in the reported depressive symptoms among the participants with various clinical conditions [χ^2^_(16)_=43.26,P<0.001]. The participants with heart disease (5.4%) reported more depressive symptoms than those with in-born heart disease (0%), kidney disease (4.3%) and other health problems (0%)(Table 4).

**Table 3:** Gender difference in reporting depressive symptoms (N=171).

PHQ_9	Female (%)	Male (%)	X^2^-value (P-value)
No depressive symptoms	56.8	77.5	15.0 (0.005)
Mild	24.7	19.1	
Moderate to severe	18.5	3.3	

**Table 4:** Different clinical conditions in reporting depressive symptoms (N=171).

PHQ_9	Heart disease	Heart disease (inborn)	Kidney disease	Other health Problem	X^2^-vaiue (P-value)
No depressive symptoms	76.0	100.0	78.3	85.7	43.26 (0.001)
Mild	18.7	0.0	17.4	14.3	
Moderate to severe	5.4	0.0	4.3	0.0	

### PTSD, depression and physiological measures

The participants with CVD who reported having PTSD symptoms had significant SBP and HR changes [t_(167)_=7.49,P<0.001 and t_(167)_=2.25,P=0.026, respectively] (Table 5). The participants with PTSD symptoms had greater SBP changes (M= 17.82,SD=11.04) than those without (M=5.22,SD=9.02). The participants with PTSD symptoms also had greater HR changes (M=7.34,SD=12.69) than those without. The participants without PTSD symptoms had smaller HR changes (M=3.0,SD=10.32) than their counterparts. There was a significant difference in the reported sleeping duration [t_(167)_=2.43,P=0.016] (Table 5). The participants who reported PTSD symptoms had a shorter sleeping duration (M=6.43,SD=2.02) than those who did not (M=7.17,SD=1.62). There was no significant difference in the health behaviours or other measures including physical activities, drinking, smoking, BMI between the participants with and without PTSD symptoms. Similar results were found in all physiological measures between the participants with and without depressive symptoms.

PTSD symptoms and trauma exposure were associated with physical health problems.

The ANOVA showed a significant relationship between the number of trauma exposures and the number of physical health problems reported [F_(15,116)_=2.22,P=0.009]. There was also a significant relationship between PTSD symptoms and the number of physical health problems reported [F_(52,67)_=1.96,P=0.005]. The traumatised participants (M=5.75,SD=3.13) reported more physical health problems than non-traumatised participants (M=3.33,SD=2.31) [t_(124)_=3.36,P=0.001]. The traumatised participants reported 1.202 times (95%CI= 1.111–1.300) more physical health problems than non-traumatised participants. The participants with PTSD symptoms (M=7.57,SD=3.55) were more significantly likely to report physical health problems than those without (M=4.79, SD=2.70) [t_(121)_=4.44,P<0.001]. Participants with PTSD symptoms were 1.29 times (95%CI= 1.18–1.43) more likely to report physical health problems than those without.

**Table 5:** Patients with cardiovascular disease with and without PTSD symptoms in reporting physiological changes (N=171).

	With PTSD	Without PTSD	t-value (P-value)
Changes Systolic Blood Pressure Diastolic Blood Pressure Heart Rate Sleep duration	17.82(11.04) 5.65(11.07) 7.34 (12.69) 6.43(2.02)	5.22 (9.02) 1.42 (11.31) 3.0 (10.32) 7.17 (1.62)	7.49 (<0.001) 1.98 (0.053) 2.25 (0.026) 2.43 (0.016)

### PTSD symptoms in relation to physiological measures

The repeated-measure ANOVA revealed that the mean SBP significantly differed between the participants with and without PTSD symptoms across the three time points [F_(1.85,303.23)_=25.71,P<0.001] (Table 6). Posthoc pairwise comparison with Bonferroni correction showed that the SBP significantly increased from T1 to T2 (mean score=120.43 vs 131.86) and significantly dropped at T3 (mean score=121.8,P<0.001). However, the increase in the SBP was not significant from T1 to T3 (mean score=120.43 vs 121.8,P=0.18). These results indicated a significant time effect on PTSD symptoms based on the SBP

The repeated-measure ANOVA showed that the mean diastolic blood pressure (DBP) significantly differed across the three time points [F_(1.96,320.67)_=4.27,P=0.015] (Table 6). Post hoc pairwise comparison with Bonferroni correction demonstrated that the DBP significantly increased from T1 to T2 (mean score=76.84 vs 79.43,P=0.031). However, the difference in the DBP between T2 and T3 did not reach significance (79.43 vs 77.24,P=0.081). The DBP change across the different time points between the participants with and without PTSD symptoms also did not reach significance [F_(1.96,320.67)_=0.86,P=0.422]. These results indicated a significant time effect on the DBP.

The repeated-measure ANOVA demonstrated that the mean HR differed significantly across the three time points [F_(1.96,320.75)_=21.03,P<0.001] (Table 6). Post hoc pairwise comparison with Bonferroni correction showed that the HR significantly increased from T1 to T2 (mean score=88.40 vs 92.27,P<0.001). However, the difference in the HR between T2 and T3 did not reach significance (92.27 vs 88.08,P=1.00). The HR change across the different time points between the participants with and without PTSD symptoms also did not reach significance [F_(1.96,320.75)_=3.00,P=0.052]. These results revealed a significant time effect on HR.

**Table 6:** Repeated measure for PTSD symptoms in relations to physiological measure across three time points (N=171).

PTSD Symptoms	Time 1	Time 2	Time 3	F-value (P-value)
Systolic Blood Pressure	120.43	131.86	121.8	25.71 (0.001)
Diastolic Blood Pressure	76.84	79.43	77.24	4.27 (0.015)
Heart Rate	88.40	92.27	88.08	21.03 (0.001)

## Discussion

The present study investigated the association between trauma exposure and/or PTSD and CVD. The prevalence of PTSD symptoms among the clinical sample was 25.7%. Substantial research has revealed significant incidents of PTSD caused by CVD in the past 20 years.^9,19^ This study proposed that patients with illness-induced PTSD have a higher prevalence of persistent/recurrent PTSD and a lower prevalence of remitted PTSD.

In this study, PTSD symptoms were significantly associated with elevated SBP across the different time points, suggesting sensitivity to homeostatic regulation, possibly explained by the polyvagal theory.^20^ This theory posits that PTSD symptoms have biological underpinnings and can manifest somatically. It suggests that the ventral vagal complex, part of the parasympathetic nervous system and known as the ‘social nervous system’ plays a crucial role. During safe conditions, it promotes rest, but during threats like trauma, it activates the sympathetic nervous system’s fight-or-flight response.

In most traumatising situations, when individuals cannot resolve threats through fight- or-flight responses, they activate an evolutionarily older, unmyelinated part of the vagus nerve to survive, leading to a shut-down condition and dysregulated physiological responses.^20^ This aligns with findings from a study on biomarkers for PTSD onset and maintenance, highlighting SBP as a consistent biomarker across 1-, 4-, and 12-month follow-ups post-trauma.^21^ Understanding PTSD’s aetiology is crucial for explaining the mechanism of the association between PTSD and CVD. In our sample, PTSD symptoms were shown to affect SBP suggesting this impact may manifest early in young adults.

Similar findings were noted by Stein et al.^22^ based on data from 10 countries (N=18,630), who reported hypertension starting as early as age 21 years in individuals with two or more childhood adversities, despite their majority being aged 45 years and older.^23^

The association of adverse life experiences, PTSD, and unhealthy behaviours aligns with the allostatic load theory.^24^ This theory posits that extreme stress triggers physiological responses such as increased heart rate, blood pressure, blood sugar levels, arteries constriction, and immune suppression. Subsequently, these responses can lead to stickier platelets, arterial wall damage, and weakened heart muscle,^24^ possibly explaining the elevated blood pressure and HR observed among the traumatized individuals in our study.

Physiological responses to stress are mediated by amygdala activation.^25^ Tawakol et al.^25^ examined both the physical and biochemical impacts of stress, supporting the allostatic load theory and its association with cardiovascular events. The elevated SBP observed in young patients in our study further supports the idea that elevated blood pressure not only poses a risk for CVD but may also influence PTSD onset and overall physical health. Our findings suggest that CVD rather than kidney disease, may have a stronger association with PTSD onset. Practitioners and researchers may fail to recognise a significant life-threatening disease as traumatic, misidentifying patients experiencing illness- induced PTSD.^7^ As a result, regardless of DSM- 5 classification, practitioners should remain vigilant in diagnosing PTSD in patients with life- threatening diseases, ensuring timely recognition and support.

Trauma-focused interventions are crucial for enhancing public health efforts in preventing and detecting CVD early. Professionals including paediatricians, child psychologists, social workers and educators need to recognize the sequelae of traumatic exposure in children and adolescents. Prevention strategies, such as regular monitoring and early interventions, should be prioritized for children and adolescents exposed to traumatic events.

### Limitations and strengths

The reliance on retrospective reporting of lifetime trauma is one of the study’s limitations; the number of lifetime traumas may be underreported or change over time. An assessment of the validity of adult retrospective accounts of unpleasant childhood events revealed that while the retrospective study may have a bias, it is not sufficiently significant to invalidate retrospective research.^26^

The present study did not investigate the biochemical consequences of trauma exposure and PTSD on CVD. Dysregulation of pro- inflammatory and anti-inflammatory cytokines due to stress may disrupt immune balance, potentially contributing to CVD development in trauma-exposed or PTSD-affected individuals.^24,27^ Future research should explore these biochemical mechanisms further, as this was beyond our study’s scope. Assessing blood pressure remains a quick and effective method to predict future cardiovascular events,^28^ with our study demonstrating a link between elevated blood pressure and chronic stress.

Future studies should consider incorporating additional psychological measures such as anxiety level. Notably, DSM-5 reclassified PTSD under trauma and stress-related disorder rather than anxiety disorder,^7^ the relationship between anxiety disorder and CVD risk remains less understood compared to trauma and depression. A 2010 meta-analysis of 20 studies discovered that individuals with premorbid anxiety faced higher risks of incident CVD and cardiac death among community samples and veterans over 37 years of follow-up.^27-30^ However, previous studies often did not adjust for depression, leading to mixed findings regarding the association between anxiety and depression.^30^

Despite its limitations, the current study highlights several strengths. It examines the association between PTSD with CVD in comparison with depression and other physical health problems like kidney disease, suggesting that specific physical conditions, like CVD, can trigger illness-induced PTSD. Given the high comorbidity of PTSD, anxiety and depression distinguishing psychological symptoms solely due to life-threatening physical conditions is challenging.^30^ This often results in underrecognition and inadequate treatment of illness- induced PTSD compared to traditional PTSD, potentially leading to more chronic or complex CVD.

Unlike studies focusing on a specific traumatic event, our study utilized a comprehensive range of traumatic events to demonstrate their significant impact on CVD through PTSD. This approach differs from previous studies limited to specific traumatic events and populations (e.g., veterans, physical or sexual abuse, women), limiting generalizability.^9,19,21^ Including various traumatic events enabled a robust comparison of their association strength with PTSD and CVD in our clinical sample. Moreover, our study extended existing literature by assessing blood pressure and HR during and after testing, facilitating temporal comparisons of traumatic memories with physical conditions.

## Conclusion

This study explored the enduring psychological and physical sequelae of trauma and PTSD, employing the polyvagal theory to elucidate their association with CVD. Elevated blood pressure and HR were significantly associated with trauma exposure and PTSD, while unhealthy behaviours were associated with certain traumatic events. Although causality was not established, PTSD mediated traditional CVD risk factors. Traumatic events likely influence biological processes, leading to adverse health outcomes in adulthood. The study underscores the importance of early detection of physiological measures in traumatized young adults, advocating their inclusion in health promotion and prevention programs to mitigate CVD risk. Screening for trauma exposure and PTSD symptoms in primary healthcare settings could enhance mental well-being and significantly reduce the burden of CVD, particularly among adolescents and young adults.

## Data sharing statement

Data is only available upon request.

**How does this paper make a difference in general practice?**

- This study highlights the complex relationship between trauma exposure, PTSD symptoms, elevated blood pressure and heart rate and unhealthy behaviours. Understanding this association is crucial for screening and early intervention.
- Recognising the abovementioned association can allow general practitioners to provide more holistic care to enhance patients’ overall well-being.
- Given that unhealthy behaviours are associated with specific traumatic events, treatment plans must be tailored.
- The findings can inform public health initiatives aimed at preventing and addressing the consequences of trauma. In particular, public health programmes can be designed to target at-risk populations effectively.
